# Educational value of surgical videos on YouTube: quality assessment of laparoscopic appendectomy videos by senior surgeons vs. novice trainees

**DOI:** 10.1186/s13017-019-0241-6

**Published:** 2019-05-09

**Authors:** Nicola de’Angelis, Paschalis Gavriilidis, Aleix Martínez-Pérez, Pietro Genova, Margherita Notarnicola, Elisa Reitano, Niccolò Petrucciani, Solafah Abdalla, Riccardo Memeo, Francesco Brunetti, Maria Clotilde Carra, Salomone Di Saverio, Valerio Celentano

**Affiliations:** 1 Department of Digestive, Hepato-Pancreato-Biliary Surgery, and Liver Transplantation, Henri-Mondor University Hospital, AP-HP, Université Paris Est, 51, Avenue du Maréchal de Lattre de Tassigny, 94010 Créteil, France; 2 Department of General and Colorectal Surgery, Northern Lincolnshire and Goole, Diana Princess of Wales Hospital, Scartho Rd, Grimsby, DN33 2BA UK; 3 Unit of Colorectal Surgery, Department of General and Digestive Surgery, Hospital Universitario Doctor Peset, Valencia, Spain; 4 Department of Digestive, Hepato-Pancreato-Biliary Surgery, and Liver Transplantation, Pitié-Salpêtrière University Hospital, AP-HP, Université Pièrre et Marie Curie (UPMC) et Paris-Descartes, Paris, France; 5 Department of General Surgery, Policlinico A. Rubino, Università di Bari, Bari, Italy; 6 University Paris Diderot, Paris France, Rothschild Hospital, AP-HP, Paris, France; 7 Department of Surgery, Addenbrooke’s Hospital, Cambridge University Hospitals NHS Foundation Trust, Cambridge, UK; 8 Colorectal Unit, Queen Alexandra Hospital, Portsmouth Hospitals NHS Trust, Portsmouth, UK

**Keywords:** Educational videos, Surgical training, Laparoscopic appendectomy, Resident surgeons, YouTube

## Abstract

**Background:**

To prepare for surgery, surgeons often recur to surgical videos, with YouTube being reported as the preferred source. This study aimed to compare the evaluation of three surgical trainees and three senior surgeons of the 25 most viewed laparoscopic appendectomy videos listed on YouTube. Additionally, we assessed the video conformity to the published guidelines on how to report laparoscopic surgery videos (LAP-VEGaS).

**Methods:**

Based on the number of visualization, the 25 most viewed videos on laparoscopic appendectomy uploaded on YouTube between 2010 and 2018 were selected. Videos were evaluated on the surgical technical performance (GOALS score), critical view of safety (CVS), and overall video quality and utility.

**Results:**

Video image quality was poor for nine (36%) videos, good for nine (36%), and in high definition for seven (28%). Educational content (e.g., audio or written commentary) was rarely present. With the exception of the overall level of difficulty, poor consistency was observed for the GOALS domains between senior surgeons and trainees. Fifteen videos (60%) demonstrated a satisfactory CVS score (≥ 5). Concerning the overall video quality, agreement among senior surgeons was higher (Cronbach’s alpha 0.897) than among trainees (Cronbach’s alpha 0.731). The mean overall videos utility (Likert scale, 1 to 5) was 1.92 (SD 0.88) for senior examiners, and 3.24 (SD 1.02) for trainee examiners. The conformity to the LAP-VEGaS guidelines was weak, with a median value of 8.1% (range 5.4–18.9%).

**Conclusion:**

Laparoscopic videos represent a useful and appropriate educational tool but they are not sufficiently reviewed to obtained standard quality. A global effort should be made to improve the educational value of the uploaded surgical videos, starting from the application of the nowadays-available LAP-VEGaS guidelines.

**Electronic supplementary material:**

The online version of this article (10.1186/s13017-019-0241-6) contains supplementary material, which is available to authorized users.

## Background

Acute appendicitis is the most common abdominal emergency worldwide, with a lifetime risk of 8.6% in males and 6.9% in females [1]. In more than 95% of cases, surgery is required [2]. The use of laparoscopic approach has remarkably increased in the last decades [2–6], showing improved results compared to open surgery in terms of postoperative recovery (e.g., pain, incidence of surgical site infection, length of hospital stay) [7–9]. In the USA, laparoscopic appendectomy (LA) represented the 43.3% of all appendectomy procedures in 2004 and the 75% in 2011, both in the settings of non-perforated (46.9% to 77.8%) and perforated (32.8% to. 66.6%) acute appendicitis [3].

LA is considered a basic procedure in the field of digestive surgery, and it represents one of the commonest interventions to begin surgical training in minimally invasive surgery. Indeed, this procedure can be safely carried out by surgical residents under the supervision of experienced surgeons [10, 11]. Moreover, it provides the basic knowledge of laparoscopic technique that must be achieved before performing more complex procedures [10].

To prepare for surgery, surgeons recur more and more often to surgical videos with YouTube being reported as the preferred source [12, 13]. Both senior surgeons and residents may watch online surgical videos for reviewing rarely performed surgeries, examine some technical details, and seeing how other colleagues work. Surgical videos are undoubtedly a useful and appropriate training tool for laparoscopy considering the video-based nature of the procedure and the display of the exact surgeon’s perspective of the intervention providing surgical trainees with essential information regarding anatomy and the different steps of the operation. However, the quality of surgical videos available on the World Wide Web has been recently questioned since most of them are uploaded without any peer review process or quality assessment [14–16]. Particularly on YouTube, videos are ranked on popularity, number of visualizations, and comments, which are not valid criteria when videos claim for educational purposes. Without adequate control and selection, video content may feature poor surgical techniques or critical safety violations that may not be immediately recognized, especially by novice trainees in the surgical field. As a result, useless or even misleading surgical videos circulate representing unvetted educational resources [14, 17].

To amend this phenomenon, an international multispecialty trainers and trainees expert committee has recently published a consensus statement on how to report a laparoscopic surgery video for educational purposes (LAP-VEGaS: LAParoscopic surgeryVideo Educational GuidelineS) in order to achieve high-quality educational videos that could improve surgical training now on [18].

The aim of the present study was to compare the evaluation of surgical trainees and senior surgeons of the 25 most viewed laparoscopic appendectomy videos listed on YouTube. Additionally, the video conformity to the LAP-VEGaS guidelines was assessed.

## Methods

### Study design

A comprehensive search was carried out on YouTube (https://www.youtube.com) on July 1, 2018, using the search terms “laparoscopic appendectomy” and “laparoscopic appendicectomy.” Videos were ordered by number of visualizations and the top 25 were selected based on the following criteria: videos uploaded between 2010 and 2018, live surgery recorded by laparoscopic camera, laparoscopic multiport intra-abdominal appendectomy, one LA procedure (no cartoon, schematized video, or multiple operations), videos made by professionals for professionals, patients aged > 12 years, and English language.

Three trainees in general and digestive surgery (SA, MN, PG) and three senior surgeons (> 100 hands-on LA) [19] expert in minimally invasive and emergency digestive surgery (VC, SDS, AM-P) evaluated independently and blindly the 25 selected videos concerning the surgical technical performance, the anatomical exposure, and the overall video quality and utility as educational tool. The study focused exclusively on the evaluation of public-domain videos on surgery. Thus, no ethical approval was necessary.

### Evaluation of surgical and education quality

For each selected video, we analyzed basic characteristics, educational content, surgeon’s laparoscopic performance, technical aspects, overall video quality and utility, and conformity to LAP-VEGaS guidelines (Table 1).

**Table 1:** Data extracted and parameters evaluated for each selected video

Video characteristics	Title
	Number of visualizations
	Source
	Country
	Upload date and number of days online
	Video length (min)
	Image quality (poor, good, high definition)
	Number of comments
	Number of likes
	Number of dislikes
Educational content	Presence of audio commentary
	Presence of written commentary
	Description of preoperative data (e.g., patient’s demographic, medical history, diagnostic data, imaging)
GOALS domains	Depth perception
	Bimanual dexterity
	Efficiency
	Tissue handling
	Autonomy
	Overall level of difficulty
Critical view of safety (CVS) criteria *Modified for LA*	Appendix exposure
	Mesoappendix transection
	Appendix division
Technical aspects	Patient’s positioning
	Trocar placement
Overall quality assessment	Overall video quality
	Overall video utility for trainees
LAP-VEGaS criteria	Authors information and video introduction
	Case presentation
	Demonstration of the surgical procedure
	Outcomes of the procedure
	Associated education content
	Peer-review of surgical videos
	Use of surgical video in educational curricula

To evaluate the surgeon’s laparoscopic performance, the examiners applied the Global Operative Assessment of Laparoscopic Skills (GOALS) rating instrument [20, 21], which has been validated as an assessment tool for video recordings of LA [22]. The GOALS is composed of six domains, including depth perception, bimanual dexterity, efficiency, tissue handling, autonomy, and overall level of difficulty. Each domain is assessed on a 5-point Likert scale (1 worst to 5 best).

The three domains of the critical view of safety (CVS) score, originally developed for laparoscopic cholecystectomy [23], were modified to apply for LA as appendix exposure, mesoappendix transection, and appendix division. These criteria were scored as 0 point if not visible, 1 point if partially visible, and 2 points if the video showed a complete critical view of safety. A score ≥ 5 was considered as a satisfactory completion of the CVS [15, 24].

Overall video quality was scored as good, moderate, or poor. Overall video utility as an education/training tool for LA was rated using a 5-point Likert scale (1 useless to 5 very useful). Finally, one independent examiner (NdeA) assessed the conformity of each video to the 37 items composing the LAP-VEGaS guidelines [18].

### Statistical analysis

Data analysis was performed with SPSS Statistics (Version 24 for Mac, IBM Corporation). Descriptive statistics were presented as frequencies (*n*) and percentages (%) for categorical variables and mean or median (standard deviation, range) for continuous and ordinal variables. Internal consistency between examiners was assessed through Cronbach’s alpha, where a value ≥ 0.7 was considered as acceptable. Spearman’s rho was calculated to assess the degree of correlation between performance measures. Binary logistic regression analysis was performed to identify factors associated with the overall video quality.

## Results

### Video selection process and video characteristics

**Fig. 1:**
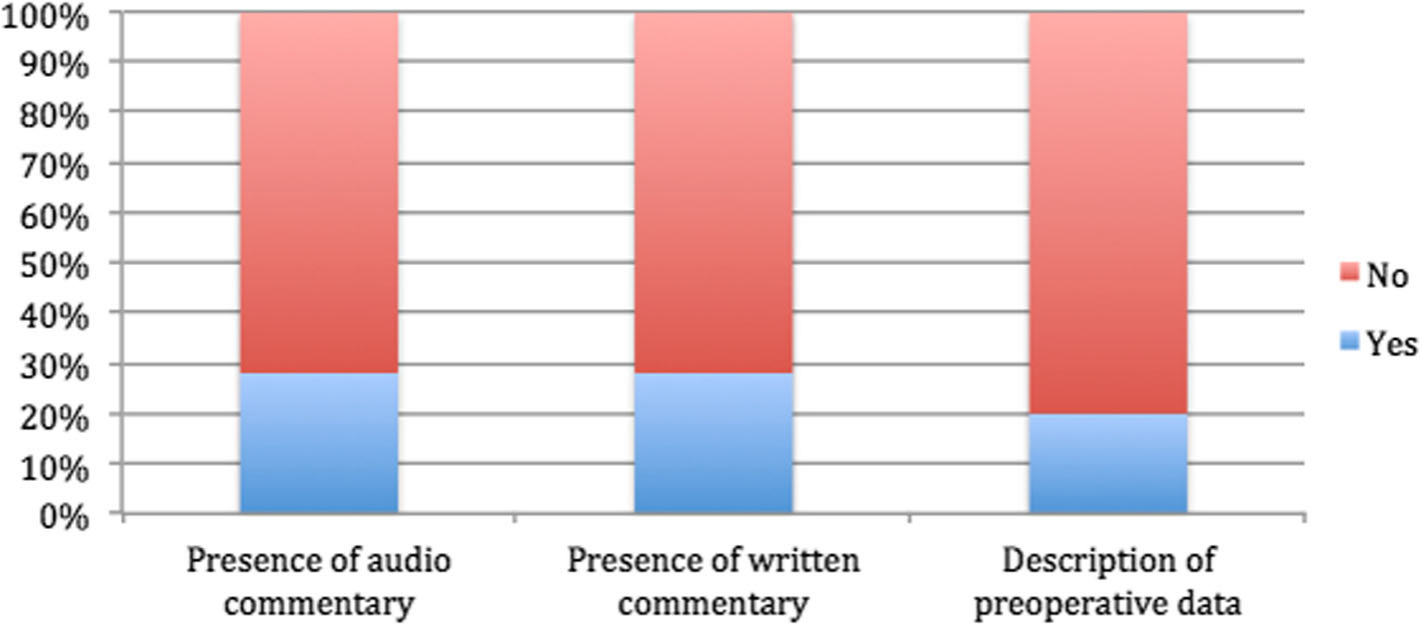
Percentage of videos presenting education contents

The search retrieved more than 31,300 videos on YouTube. Once sorted by number of views, we watched the consecutive videos to check for eligibility and we included the first most viewed 25 videos that met the predefined selection criteria. We excluded two videos that were duplicates, one video that included cartoon animations, and one video that was commented in a language other than English. The characteristics of the selected 25 videos are displayed in Table 2. Overall, six videos (24%) were made in North America, three (12%) in South America, seven (28%) in Europe, eight (32%) in Asia, and one (4%) in Oceania. The majority was made by surgeons from tertiary care hospitals/academic institutions (ten videos, 40%) or secondary care hospitals (six videos, 24%). On average, videos were available online for 1746.5 days (range 395–2767 days). The mean video length was 7.5 min (SD 5.92), ranging from 1.34 to 27.30 min. These videos received a mean of 41 comments (range 0–457), with overall more “likes” (mean 201.9; range 9–1941) than “dislikes” (mean 18.5; range 0–181). The image quality was rated as poor for nine (36%) videos, as good for nine (36%) videos, and as high definition for seven (28%) videos. The evaluation of the educational content showed that audio/written commentaries were present in 28% of cases and a detailed case description with preoperative data in only 20% of videos (Fig. 1).

**Table 2:** Characteristic of the 25 selected videos on laparoscopic appendectomy (ordered by number of visualizations on July 1, 2018)

Number	Title and Link	Number of visualizations	Source	Country	Number of days online	Length (min)	Image quality	Number of comments	Number of likes	Number of dislikes
1	Acute Appendicitis - Initial Stage - Ultracision + Endoloops https://www.youtube.com/watch?v=uYhvRl1u4ac	418,318	Secondary hospital	Brazil	1427	6.45	Poor quality	215	1000	181
2	Laparoscopic appendicectomy (appendectomy) https://www.youtube.com/watch?v=ljwa7FkGyhc	317,271	Tertiary hospital/academic institution	Australia	1161	8.53	Good quality	0	591	59
3	Appendectomy for ruptured appendicitis https://www.youtube.com/watch?v=VrvOhM9euns	298,075	Commercial institution	The USA	1797	5.2	High definition	457	1941	67
4	Laparoscopic Appendicectomy for Acute Appendicitis with Appendix Mass https://www.youtube.com/watch?v=cw-sbEoG0Eo&frags=pl%2Cwn	143,183	Private practice	The UK	2637	14.32	Poor quality	80	211	21
5	Laparoscopic Appendectomy by Advanced Surgeons PC https://www.youtube.com/watch?v=T8bdFYMIJvg	86,130	Tertiary hospital/academic institution	The USA	2254	4.14	Good quality	23	140	13
6	Latest treatment/ surgery for Acute Appendicitis - Laparoscopic Appnedectomy https://www.youtube.com/watch?v=J7IbZmqhVvU	74,728	Tertiary hospital/academic institution	India	479	5.17	Poor quality	8	80	16
7	Laparoscopic Appendectomy https://www.youtube.com/watch?v=M8RhIDOz-5U	68,464	Tertiary hospital/academic institution (SAGES)	The USA	2592	4.11	Poor quality	27	146	8
8	Laparoscopic Appendectomy - Blinddarmoperation https://www.youtube.com/watch?v=nl7Iu37SS-s	43,165	Private practice	Germany	2222	1.34	High definition	17	64	5
9	Laparoscopic Appendectomy https://www.youtube.com/watch?v=iYdUGSL006Q	41,733	Tertiary hospital/academic institution	The UK	1868	27.3	Good quality	3	36	8
10	Painful and Swollen Appendix Removal Surgery - Laparoscopic Appendectomy https://www.youtube.com/watch?v=_VK7oxWd1zg	41,217	Commercial institution	India	395	4.09	Poor quality	30	107	13
11	Laparoscopic Appendectomy Surgery Video https://www.youtube.com/watch?v=AD1TM9kf7ak	35,362	Secondary hospital	India	1365	3.33	High definition	1	116	11
12	Perforated Appendicitis - Fecalith on the Base - Hook + Endoloops https://www.youtube.com/watch?v=R9w_6F4hzD0&frags=pl%2Cwn	34,799	Secondary hospital	Brazil	1731	13.31	Good quality	34	124	9
13	Laparoscopic Appendectomy - Monopolar Hook and Endoloops - 1080p + GoPro https://www.youtube.com/watch?v=cmODAlhMO0k	32,823	Secondary hospital	Brazil	689	20.14	High definition	45	120	10
14	Laparoscopic Appendicectomy High Definition Video by Dr. R.K. Mishra https://www.youtube.com/watch?v=AwRCrcifI70	29,819	Secondary hospital	India	2174	5.21	Good quality	17	81	6
15	APPENDICITIS-Keyhole Surgery- 5 min demo (Laparoscopic Appendectomy) https://www.youtube.com/watch?v=EP7q0tnTdDw	27,658	Tertiary hospital/academic institution	The UK	2018	5.02	Good quality	36	55	7
16	Laparoscopic Appendectomy https://www.youtube.com/watch?v=IG-uQUSyGC8	18,195	Private practice	India	1733	7.27	High definition	5	35	9
17	How to do a laparoscopic appendicectomy https://www.youtube.com/watch?v=18eYVp244mQ	14,321	Tertiary hospital/academic institution	The UK	1662	6.54	Good quality	2	84	6
18	Lap. Appendectomy (unedited-08)-Recurrent appendicitis ligating the appendix with endo loop https://www.youtube.com/watch?v=uwSLOjwHTdY	14,318	Tertiary hospital/academic institution	Bangladesh	1672	11.29	Good quality	6	15	3
19	laparoscopic appendectomy standard technique (real-time) https://www.youtube.com/watch?v=4vfv5kE_sRo	10,261	Private practice	Russia	2281	9.41	Poor quality	1	14	1
20	Lap appendectomy - Removal of a retrocecal vermiform appedix. https://www.youtube.com/watch?v=_O4zjJ-RKpU	10,101	Private practice	Greece	2537	6.57	Good quality	3	18	0
21	Laparoscopic appendectomy https://www.youtube.com/watch?v=x8sUeH5M5Q0	9091	Unknown	The USA	574	5.37	High definition	7	27	0
22	Laparoscopic Appendectomy. An Improvised method. https://www.youtube.com/watch?v=9kb0ibKl1jE	8726	Unknown	The UK	2767	5.11	Poor quality	3	9	1
23	Laparoscopic Appendectomy at The Mount Sinai Hospital https://www.youtube.com/watch?v=gJ5U-b32jhc	7628	Tertiary hospital/academic institution	The USA	1877	2.48	High definition	2	15	5
24	Laparoscopic Appendectomy Easy Steps 6 KG Hospital Bangladesh https://www.youtube.com/watch?v=hpkuxIaiIi8	7581	Secondary hospital	Bangladesh	2257	3.03	Poor quality	1	11	1
25	Laparoscopic Appendectomy https://www.youtube.com/watch?v=vJT09sJKcM4	6500	Tertiary hospital/academic institution (SAGES)	The USA	1494	3.01	Poor quality	2	9	3

### GOALS and CVS assessment

The detailed GOALS assessment is reported in Table 3. The displayed scores for each domain represent the overall score obtained by consensus among the three senior surgeons vs. the three trainees. The Cronbach’s alpha was poor to moderate for the domains depth perception, bimanual dexterity, efficiency, tissue handling, and autonomy. Conversely, it was very good for the evaluation of the overall level of difficulty. The internal consistency among senior examiners ranged between 0.508 and 0.958 whereas among trainees it ranged between 0.331 and 0.961.

**Table 3:** GOALS assessment of the 25 selected videos on laparoscopic appendectomy

Video no.	Depth perception		Bimanual dexterity		Efficiency		Tissue handling		Autonomy		Overall level of difficulty	
	Seniors	Trainees	Seniors	Trainees	Seniors	Trainees	Seniors	Trainees	Seniors	Trainees	Seniors	Trainees
1	1	4	5	5	3	4	2	2	2	4	1	1
2	2	4	3	4	4	5	3	4	5	5	1	1
3	3	5	3	5	4	5	2	2	3	5	3	3
4	3	4	4	5	3	4	4	5	4	5	4	4
5	4	5	4	5	3	5	2	5	4	5	3	2
6	4	5	4	5	5	5	4	4	3	5	3	2
7	3	5	3	5	3	5	3	4	4	5	1	1
8	3	3	2	4	4	4	3	4	4	5	1	1
9	4	3	2	5	2	3	2	2	2	3	1	1
10	2	3	3	5	2	4	3	3	4	5	1	2
11	3	3	5	4	3	5	2	4	4	4	1	1
12	3	3	3	3	5	5	3	5	5	4	4	4
13	1	3	4	3	3	4	3	5	3	5	1	1
14	5	3	4	5	4	3	3	2	2	5	1	1
15	2	4	5	5	4	4	2	4	4	4	1	1
16	3	3	4	5	5	5	3	3	4	4	1	1
17	3	4	3	4	5	3	4	5	4	5	1	1
18	3	3	4	5	3	5	4	4	4	4	1	1
19	5	4	3	5	5	4	3	3	4	4	1	1
20	4	4	3	4	3	5	3	5	4	5	2	3
21	5	3	3	5	2	5	3	3	4	4	1	2
22	3	3	4	4	3	5	3	4	4	4	1	1
23	5	5	4	5	3	4	3	3	4	5	3	3
24	4	4	4	5	3	4	3	4	4	5	1	1
25	4	5	3	5	1	4	3	3	4	5	1	2
Cronbach’s *α*	0.315		0.218		0.132		0.530		0.284		0.937	

**Fig. 2:**
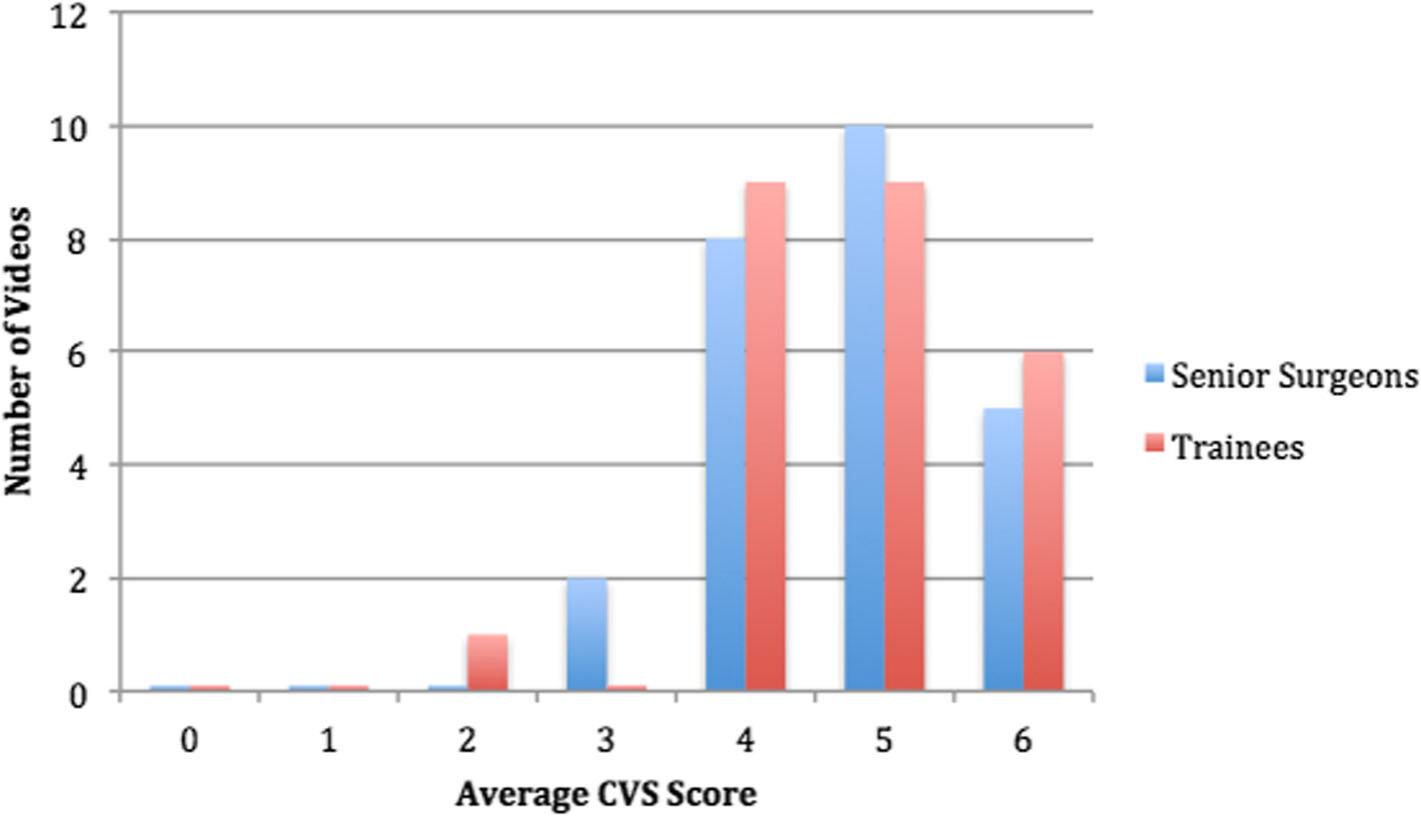
Distribution of critical view of safety (CVS, modified for LA) scores for the selected 25 videos as evaluated by senior surgeons and trainees

The median total CVS score was 5 for both senior and trainee examiners. The distribution of the average CVS scores for the selected 25 videos is shown in Fig. 2. Fifteen videos (60%) demonstrated a satisfactory CVS score (≥ 5) as scored by senior surgeons or trainees with a 52% concordance rate. For the total CVS score, the consistency between the examiners was good, with a Cronbach’s alpha of 0.777 for the three senior examiners and of 0.823 for the three trainees. Among all examiners, the internal consistency was found at 0.691. The highest consistency was observed for the domain “mesoappendix transection,” with a Cronbach’s alpha of 0.882 and 0.859 for senior and trainee examiners respectively.

### Technical aspects

There was a 100% agreement among examiners for the patient’s positioning evaluation. It was correctly described in 4 videos (16%) and not shown in 21 (84%). Concerning the trocars’ placement, there was a 100% agreement among senior examiners: 4 videos (16%) showed a correct trocar positioning, 6 (24%) an incorrect, and 15 videos (60%) did not show it. Among trainee examiners, the consistency was good (Cronbach’s alpha 0.830), although they do not agree on all videos.

### Overall video quality and utility

Video quality was scored as good, moderate, or poor. Results are displayed in Table 4. Overall, a 100% agreement was found for only four videos (one rated as good, one rated as moderate, and two rated as poor quality videos). Agreement among senior surgeons was higher (17/25 videos (68%) scored exactly the same by all three examiners, Cronbach’s alpha 0.897) than among trainees (8/25 videos (32%) scored exactly the same by all three examiners, Cronbach’s alpha 0.731).

**Table 4:** Overall video quality assessment (good, moderate, or poor) by senior surgeons and novice trainees in digestive surgery

Number	Senior surgeons			Novice trainees		
	Video quality examiner 1	Video quality examiner 2	Video quality examiner 3	Video quality examiner 4	Video quality examiner 5	Video quality examiner 6
1	Moderate	Moderate	Poor	Moderate	Poor	Moderate
2	Good	Good	Good	Good	Good	Good
3	Moderate	Moderate	Moderate	Good	Good	Good
4	Moderate	Moderate	Good	Poor	Moderate	Moderate
5	Moderate	Moderate	Moderate	Moderate	Moderate	Moderate
6	Poor	Poor	Moderate	Moderate	Poor	Poor
7	Moderate	Moderate	Moderate	Moderate	Poor	Moderate
8	Poor	Poor	Moderate	Good	Poor	Poor
9	Poor	Poor	Poor	Poor	Moderate	Moderate
10	Poor	Poor	Poor	Poor	Poor	Poor
11	Moderate	Moderate	Moderate	Moderate	Moderate	Poor
12	Moderate	Moderate	Moderate	Moderate	Good	Moderate
13	Moderate	Moderate	Moderate	Good	Good	Moderate
14	Poor	Poor	Poor	Moderate	Moderate	Moderate
15	Moderate	Moderate	Moderate	Poor	Moderate	Poor
16	Poor	Poor	Good	Moderate	Good	Moderate
17	Good	Good	Good	Good	Good	Moderate
18	Moderate	Moderate	Good	Good	Good	Moderate
19	Poor	Poor	Poor	Poor	Good	Moderate
20	Poor	Poor	Moderate	Good	Good	Moderate
21	Moderate	Moderate	Moderate	Good	Good	Moderate
22	Poor	Poor	Poor	Poor	Poor	Moderate
23	Poor	Poor	Poor	Moderate	Moderate	Moderate
24	Poor	Poor	Poor	Poor	Poor	Poor
25	Poor	Poor	Poor	Moderate	Moderate	Moderate

**Fig. 3:**
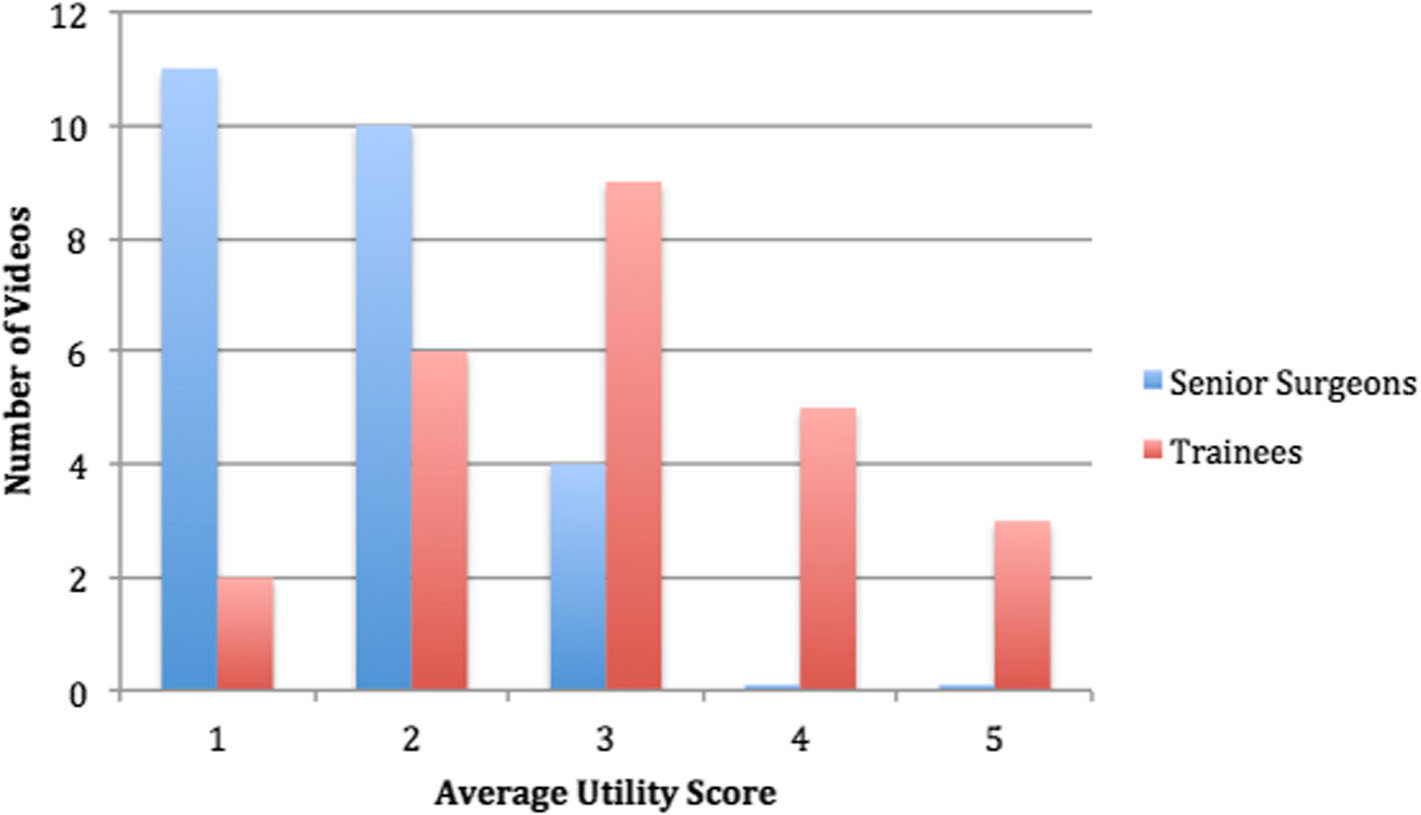
Distribution of overall video utility scores for the selected 25 videos as evaluated by senior surgeons and trainees

The mean overall videos utility was 1.92 (SD 0.88) for senior examiners and 3.24 (SD 1.02) for trainee examiners. The distribution among the different categories is shown in Fig. 3. Consistency was very good for senior surgeons (Cronbach’s alpha 0.915) and acceptable for trainee surgeons (Cronbach’s alpha 0.740).

### LAP-VEGaS conformity

The LAP-VEGaS evaluation showed that all videos reported the surgical procedure in a step-by-step fashion (LAP-VEGaS item 17), and for all of them, the number of views and comments were available (LAP-VEGaS item 37). For 52% of videos, an audio or written commentary was provided in English (LAP-VEGaS item 26). However, the majority of the LAP-VEGaS items (*n* = 24, 64.8%) were found in no video. The conformity to the LAP-VEGaS guidelines was very weak, with a median value of 8.1% (range 5.4–18.9%). The highest percentage of conformity was observed for videos #3, 4, and 17 (18.9%) (Additional file 1: Table S1). There was a positive correlation between the percentage of conformity to LAP-VEGaS and the number of likes (rho 0.691; *p* < 0.0001) and dislikes (rho 0.639; *p* = 0.001).

### Factors associated with overall video quality

Based on senior surgeons’ assessment only, we divided the selected videos into two groups: moderate/good quality (*n* = 13) vs. poor quality (*n* = 12) videos. For 17/25 videos (68%), there was a 100% agreement among the three senior examiners. The remaining consensus was reached by discussion and a final grade (moderate-good or poor quality) was attributed to the video. Then, we used binary logistic regression to evaluate the association between overall video quality and several video characteristics. The number of likes, the presence of audio/written, commentary, the utility score, and the LAP-VEGaS conformity were significantly associated with the probability of rating the video as moderate/good (Table 5).

**Table 5:** Factors associated with overall video quality based on senior surgeon assessment

	Moderate/good quality videos (*n* = 13)	Poor quality videos (*n* = 12)	*p* value Binary logistic regression	Odds ratio
Number of visualizations [median(range)]	35,362 (9091–4,183,318)	14,228 (6500–74,728)	0.148	
Number of days online [median(range)]	1672 (574–2637)	2025.5 (395–2767)	0.552	
Length (min) [median(range)]	6.45 (3.33–24.14)	5.14 (1.34–27.30)	0.494	
Number of comments [median(range)]	27 (0–457)	3 (1–30)	0.074	
Number of likes [median(range)]	124 (15–1941)	26.5 (9–107)	0.019	1.029 (1.00–1.05)
Number of dislikes [median(range)]	10 (0–181)	5 (0–16)	0.170	
CVS score ≥ 5 [*n* (%)]*	10 (76.9)	5 (41.7)	0.111	
GOALS score ≥ 20 [*n* (%)]*	8 (61.5)	6 (50)	0.695	
Utility score [mean(SD)]	2.51 (0.68)	1.27 (0.58)	0.006	2.50 (2.35–17.95)
LAP-VEGaS conformity (%)[mean(SD)]	12.89 (4.95)	6.76 (2.44)	0.014	1.15 (1.08–2.11)
Presence of audio/written commentary	10 (76.9)	3 (25)	0.014	3 (1.59–6.5)
Description of preoperative data [*n* (%)]	5 (38.5)	0	0.999	
Image quality			0.364	
• Good	6(46.2)	3 (25)		
• Poor	3(32.1)	6 (50)		
• High definition	4(38.8)	3 (25)		

*CVS* critical view of safety, *GOALS* Global Operative Assessment of Laparoscopic Skills, *LAP-VEGaS* laparoscopic surgery video educational guidelines

*Calculated on the mean of the three senior surgeons’ assessment

## Discussion

The present study reports a detailed quality evaluation of the most viewed 25 surgical videos on LA available on YouTube on July 1, 2018. These videos were available online for a mean of 4.7 years and were watched more than one million times by people worldwide. Considering the tremendous spread, it is reasonable for the scientific community to verify the educational value of these public domain e-learning tools.

First of all, we objectivized that the image quality of the uploaded videos is very heterogeneous: the most viewed video was rated as poor image quality; 50% of the ten most viewed videos were of a poor image quality. Surprisingly, the image quality did not influence the popularity of the videos although it appears essential in laparoscopic surgery in 2018 to have a high definition image to achieve efficiency [25, 26]. Moreover, essential technical aspects, such as the description of patient’s or trocars positioning, and educational content, like audio/written commentary and formal case presentation, were missing in the large majority of the evaluated videos. This is also a pitfall for videos with educational purposes. Indeed, it appears crucial to describe demographic patient’s characteristics, such as body mass index and comorbidity, which may influence the surgical set-up and the surgical difficulty [18, 27].

To assess laparoscopic skills and safety on the videos, the three senior surgeons and three trainees applied GOALS and CVS scores. Senior examiners evaluated the surgeon’s laparoscopic proficiency as moderate (only 56% of video had a GOALS score > 20) with an adequate critical view of safety in 60% of cases. Trainees tended to overscore the surgeon’s proficiency in laparoscopy (GOALS score > 20 for 95% of videos) but they agreed on the CVS assessment. Overall, the level of difficulty of the displayed LA procedures was judged as low by both senior surgeons and trainees, with a very good agreement. This may not surprise considering the type of basic intervention that LA represents in general and digestive surgery and the selection that surgeons who uploaded their videos may do in order to share online only their best cases.

The overall video quality was highly heterogeneous as well, as judged by senior surgeons or trainees. Only four videos (16%) were evaluated as poor, moderate, or good unanimously. Although the inter-examiner agreement was acceptable, this indicates how difficult is to judge the quality of a surgical video without a specific rating system. The same can be said for the overall video utility as an educational tool. In this case, trainees found the videos much more useful than senior surgeons, as expected by the lower level of experience (and the eager to learn) and the lower capacity to correctly evaluate the surgical technique. However, this also claims for caution in the use of popular domain videos as e-learning instruments for LA, as observed for other laparoscopic general surgery procedures, including laparoscopic cholecystectomy, fundoplication, or right hemicolectomy [13–17, 28]. Rodriguez et al. [14] recently evaluated the top 10 YouTube videos on laparoscopic cholecystectomy. They found that those videos showed suboptimal technique with frequent potentially dangerous safety violations. They warned about the low quality of the most popular YouTube videos and claimed for the dissemination of high-quality educational content by surgical societies or formal educational platforms. The same key message is read in the article of Deal et al. [15] that evaluated 160 short videos on laparoscopic cholecystectomy and found a low frequency of CVS, an average GOALS technical performance and no correlation between the number of views or likes and a higher video quality. In the present study, we observed that the number of likes was significantly associated with a moderate/good video quality, together with the mean utility score and the presence of audio/written commentary. However, it appears evident that the discrepancy in video quality may not be easily recognized by viewers, especially novice trainees or non-professionals, who may base their selection criteria on popular web-indices, such as the number of visualizations rather than surgical quality and veracity [14, 27]. To date, most uploaded videos, especially on YouTube, do not undergo a standardized peer-review process. This is basically unregulated, and valuable videos accredited by scientific societies may not appear in top ranked list. Indeed, the video source may be related to the video quality, authenticity, and reliability. Some studies observed that videos uploaded by tertiary care/academic centers [16] or industrial sources [28] have a higher educational value and global video quality score. However, this raises another important issue concerning public domain surgical videos. Most of the time, the sponsoring or funding source is not declared. Academic institution may upload videos on YouTube for primary educational purposes, which may not be the case for industries and companies selling surgical devices or materials [27]. Moreover, sponsored videos may be of better image quality (high definition), resolution, montage, and editing, thus resulting in an overall better evaluation by viewers even if delivering misleading or not-evidence based information.

As laparoscopic videos are widely considered as a useful adjunct to operative training but most of them are found deficient in many aspects to be considered as an educational tool, the LAP-VEGaS guidelines on reporting a laparoscopic surgery video for educational purposes were published in 2018 [18]. We applied, for the first time in our knowledge, these 37-item guidelines to the selected videos in order to assess, a posteriori, the rate of conformity to what is considered nowadays the standard of quality. Indeed, the average conformity rate was very low (8%). However, we found that a higher LAP-VEGaS conformity percentage was significantly associated with an overall moderate/good video quality, indicating that by applying these guidelines we can expect to drastically increase the quality of the uploaded videos in laparoscopic surgery.

There are limitations in this type of study. We analyzed laparoscopic videos available on YouTube only. This is reported as the most popular video source, especially among surgical residents [12], but there is a variety of alternative sources, both free-access and pay-per-view, that needs to be explored (e.g., social media platforms, formal educational websites). Although we performed a comprehensive search and we focused on a single surgical procedure, this may not be easily replicated because videos on YouTube are continuously uploaded and removed. It must be noted that there may be many different reasons for surgeons to upload their videos on YouTube, not necessarily for educational purposes. However, once they became freely available, they will be very likely viewed for training; thus, a more conscientious video upload is warranted.

## Conclusion

Videos of laparoscopic surgery represent a useful and appropriate educational tool in digestive and general surgery, which should be implemented in the operative training. Recurring to public domain videos, most often on YouTube, is widespread and currently not regulated. Thus, a global effort should be made to improve the educational value of the uploaded surgical videos, starting from the application of the nowadays-available LAP-VEGaS guidelines.

## Additional file

Additional file 1: **Table S1.** Conformity to the 37 items of the LAP-VEGaS guidelines. (DOCX 172 kb)

## Abbreviations

CVS: Critical view of safety
GOALS: Global Operative Assessment of Laparoscopic Skills
LA: Laparoscopic appendectomy
LAP-VEGaS: LAParoscopic surgeryVideo Educational GuidelineS
